# Aortic Dissection and Postpartum Cardiomyopathy in a Postpartum Young Woman: A Case Report Study

**DOI:** 10.5812/ircmj.9849

**Published:** 2014-04-05

**Authors:** Rozita Jalalian, Mehrdad Saravi, Bahar Banasaz

**Affiliations:** 1 Department of Cardiology, Babol University of Medical Sciences, Babol, IR Iran; 2 Department of Internal Medicine, Babol University of Medical Sciences, Babol, IR Iran

**Keywords:** Aortic Aneurysm, Familial Thoracic 1, Cardiomyopathies, Peripartum Period, Women

## Abstract

**Introduction:**

Aortic dissection is a rare condition in young women and usually related with congenital anomalies of aorta and connective tissue disorders. We reported a 34-year-old postpartum woman with aortic dissection.

**Case Presentation:**

The patient complained of respiratory distress and weakness with no abdominal pain or chest pain 20 days after delivery and had no history of hypertension during pregnancy and perinatal or prior heart disease. Postpartum cardiomyopathy and left ventricular dysfunction were diagnosed by imaging study and cardiac enzyme level. Finally, CT-scan was performed and showed aortic dissection. The patient underwent surgery and after surgery, she was alive without any problem.

**Conclusions:**

Patients with peripartum cardiomyopathy and aortic dissection could be cured with good medical care.

## 1. Introduction

Only 60% to 70% of patients with aortic dissection could be accurately diagnosed in the early stage of hospital admission (1, 2). Acute aortic dissection may occur in pregnancy in association with severe hypertension due to preeclampsia, coarctation of aorta or connective tissue disorders such as Marfan's syndrome (3). Patients typically present with severe chest pain or interscapular pain associated with end organ ischemia and/or acute heart failure secondary to acute aortic incompetence or hemopericardium and tamponade (3), but when associated with left ventricular dysfunction clinical manifestation of dissection might be masked. We reported a young woman with acute aortic dissection associated with left ventricular dysfunction due to peripartum cardiomyopathy.

## 2. Case Presentation

**Figure 1.**
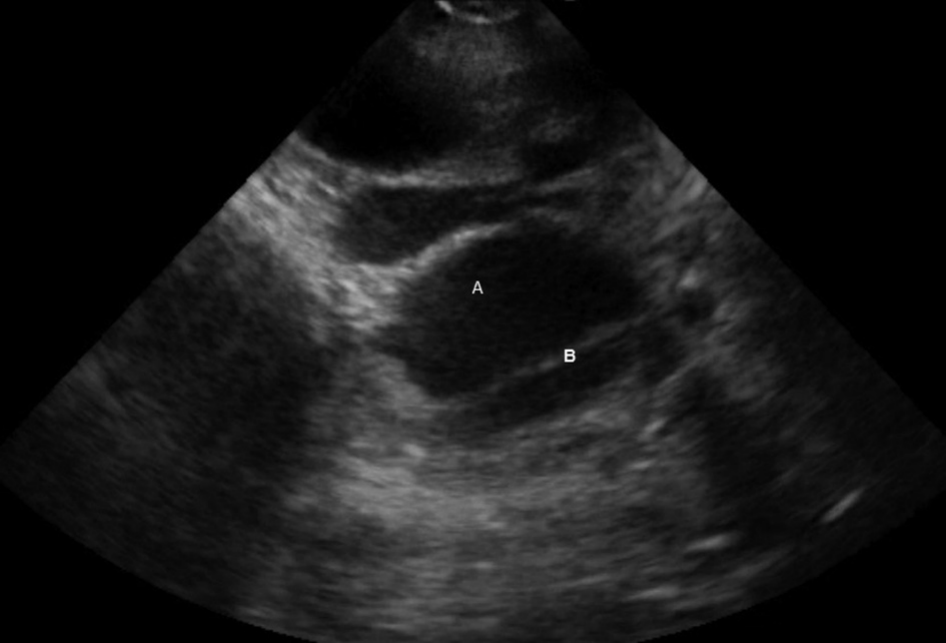
A, Descending Thoracic Aorta (short axis); B, Intimal Flap

**Figure 2.**
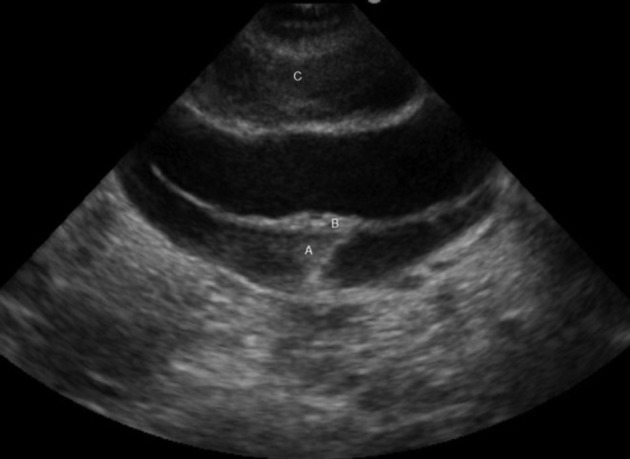
A, False Lumen and Thrombus; B, Intimal Flap; C, Periaortic Hematoma

**Figure 3.**
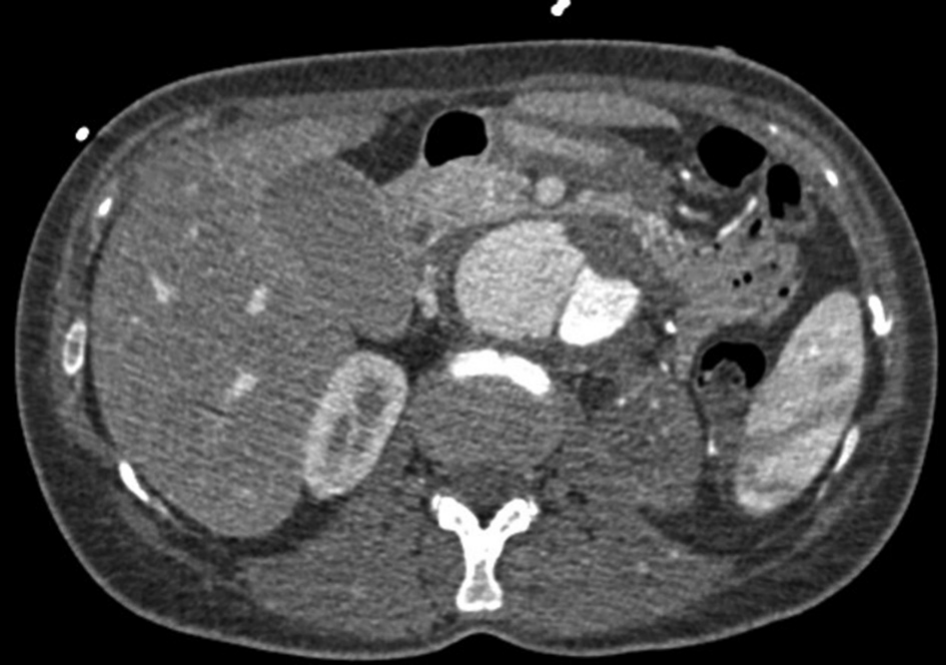
Axial Contrast CT-Scan

A 34-year-old female, gravid 1 and para 1, was admitted to emergency ward of Ayatollah Rohani hospital, Babol, Iran in august 2012, 20 days after delivery of her baby with cesarean section. She experienced dyspnea and respiratory distress for 2 days before admission, which increasing from the last 3-4 hours. Around one week after cesarean section, she experienced a very sharp abdominal pain attributed to cesarean section. In physical examination, we just found fine crackles in the right lung and decreased breath sound in the left lung and she described feeling weakness and there was no Marfanoid appearance or other appearances related with aortic dissection. Her blood pressure was 150/80 mmHg, body temperature was 37.5ºC, pulse rate of 118 beats/min and respiratory rate of 30 breaths/min. There was no tenderness, distention of abdomen or rebound pain. Hemoglobin level was 10.4 gr/dL, WBC 12500 with neutrophil count of 88%, platelet 250000, CK-MB 28 U/l (normal< 24), erythrocyte sedimentation rate 68 mm/hr (normal< 20 mm/hr), c-reactive protein (CRP) 3+. Work-up was focused on heart problem. Pro BNP level was 25000 pg/mL (normal < 125) and D-dimer was 4+. Electrocardiogram data showed sinus tachycardia, left anterior hemi block and LVH. Chest X-ray showed left pleural effusion and dilated aortic arch and descending aorta. Transthoracic echocardiography (Sonosite, M-turbo) revealed severe LV systolic dysfunction with ejection fraction of 20-25%, mild dilated aortic root and ascending aorta (4.5 cm), mild to moderate aortic insufficiency, massive left side pleural effusion, initial flap extended from proximal part of descending thoracic aorta to abdominal aorta, abdominal aorta was aneurismal (up to 7 cm) with intimal flap and hematoma and fresh thrombus in false lumen with periaortic hematoma (Figures 1 and 2). Axial contrast enhanced CT-scan and angiography were performed (Multi detector 16 slice CT-angiography Siemens company) and revealed extension of intimal flap that separated true from false lumen from left subclavian artery to descending thoracic and abdominal aorta with periaortic hemorrhage surround dissection (dissection, Stanford's type A) (Figure 3). Peripartum cardiomyopathy and descending aortic dissection were diagnosed. Due to high-risk situation (LV dysfunction and extensive dissection), surgical and intervention procedures had been postponed by multiple surgical and interventional groups for 45 days. An experienced surgeon and interventional teams accepted the responsibility of treatment.

Patient underwent hybrid operation with surgical repair of thoracoabdominal aorta, left common carotid, left subclavian artery, descending thoracic aorta, superior mesenteric artery and right iliac artery (Shariati Hospital, Tehran, Iran). Interventional procedure was performed with stents and grafts of descending thoracic and both iliac and abdominal aorta. Thirty days after surgery she was alive without any serious problem (Boxes 1 and 2).

**Box 1.** Characteristics of the Patient

A 34-year-old female
**Gravid 1 para 1**
**Underwent cesarean section 20 days before**
**Referred with dyspnea and respiratory distress**
**Sharp abdominal pain 7 weeks earlier **
**Fine crackles in the right lung**
**Decreased breath sound in the left lung**
**Blood pressure was 150/80 mm Hg, 118 beats/min, respiratory rate 30 breaths/min**

**Box 2.** Paraclinic Finding

Pro BNP: 25000 pg/mL and D-dimer:4+
**Electrocardiogram**
sinus tachycardia, left anterior hemiblock and LVH
**Chest X-ray**
pleural effusion, dilated aortic arch and descending aorta
**Transthoracic echocardiography**
sever LV systolic dysfunction, EF:20-25%, mild dilated aortic root and ascending aorta, intimal flap from descending thoracic aorta to abdominal aorta, abdominal aorta was aneurismal
**CT-angiography**
Intimal flap from false lumen from subclavian to descending and adnominal aorta

## 3. Discussion

Acute aortic dissection is a sudden event as abrupt longitudinal and circumferential separation of the media of the aorta, resulting in blood leaking from the true lumen into a false lumen through a tear, usually small, in the intima (4) and is one of the most life-threatening cardiovascular insults. Untreated acute aortic dissection has an estimated mortality rate of 1 to 2% per hour during the first 24 to 48 hours of onset (5). However, owing to its diverse clinical manifestation, acute aortic dissection is accurately and promptly diagnosed in only about 60% of cases (5). Hsieh et al. (5) reported a 28-year old young female admitted to emergency room one week after delivery with no history of any cardiac diseases presented with mild transient back pain with sweating followed by periumbilical cramping pain, vomiting and general malaise. Echocardiography revealed global left ventricle hypokinesis with borderline performance and computed tomography showed abnormal mediastinal shadow and aortic contour but at the end, the patient died. However, aortic dissection is very rare in young females and in association with severe hypertension due to preeclampsia, coarctation of the aorta or connective tissue disorders as Marfan syndrome, congenital heart disease, trauma and inflammatory diseases (3), but our patient had no obvious predisposing factor for aortic dissection. Expect Hsieh (5) we could not find any article reporting a patient with peripartum cardiomyopathy and acute aortic dissection. Peripartum cardiomyopathy is an idiopathic heart failure occurring in the absence of any determinable heart disease during the last month of pregnancy or the first 5 months postpartum (6). The incidence of peripartum cardiomyopathy is less than 1% in the world (7-10). Aortic dissection has a low incidence (11) per se, therefore combination of these two conditions is extremely rare.

The clinical aspects most commonly associated with acute aortic dissection include history of hypertension, syncope or pain (4); but neither was seen in our patient. In our patient, like Hsieh report, elevated serum levels of cardiac enzyme and C-reactive protein prompted us to attribute the pathogenesis of aortic dissection to cardiomyopathy with left ventricular dysfunction, despite the fact that these markers could also be elevated in patients with acute aortic dissection (12). This was an interesting case because of concordance of both peripartum cardiomyopathy and extensive dissection of aorta, which was finally treated successfully.
